# Costs of Eliminating Malaria and the Impact of the Global Fund in 34 Countries

**DOI:** 10.1371/journal.pone.0115714

**Published:** 2014-12-31

**Authors:** Brittany Zelman, Anthony Kiszewski, Chris Cotter, Jenny Liu

**Affiliations:** 1 The Global Health Group, University of California San Francisco, San Francisco, CA, United States of America; 2 Bentley University, Waltham, MA, United States of America; Tulane University School of Public Health and Tropical Medicine, United States of America

## Abstract

**Background:**

International financing for malaria increased more than 18-fold between 2000 and 2011; the largest source came from The Global Fund to Fight AIDS, Tuberculosis and Malaria (Global Fund). Countries have made substantial progress, but achieving elimination requires sustained finances to interrupt transmission and prevent reintroduction. Since 2011, global financing for malaria has declined, fueling concerns that further progress will be impeded, especially for current malaria-eliminating countries that may face resurgent malaria if programs are disrupted.

**Objectives:**

This study aims to 1) assess past total and Global Fund funding to the 34 current malaria-eliminating countries, and 2) estimate their future funding needs to achieve malaria elimination and prevent reintroduction through 2030.

**Methods:**

Historical funding is assessed against trends in country-level malaria annual parasite incidences (APIs) and income per capita. Following Kizewski et al. (2007), program costs to eliminate malaria and prevent reintroduction through 2030 are estimated using a deterministic model. The cost parameters are tailored to a package of interventions aimed at malaria elimination and prevention of reintroduction.

**Results:**

The majority of Global Fund-supported countries experiencing increases in total funding from 2005 to 2010 coincided with reductions in malaria APIs and also overall GNI per capita average annual growth. The total amount of projected funding needed for the current malaria-eliminating countries to achieve elimination and prevent reintroduction through 2030 is approximately US$8.5 billion, or about $1.84 per person at risk per year (PPY) (ranging from $2.51 PPY in 2014 to $1.43 PPY in 2030).

**Conclusions:**

Although external donor funding, particularly from the Global Fund, has been key for many malaria-eliminating countries, sustained and sufficient financing is critical for furthering global malaria elimination. Projected cost estimates for elimination provide policymakers with an indication of the level of financial resources that should be mobilized to achieve malaria elimination goals.

## Introduction

Since 1900, 113 countries have eliminated malaria and 34 are currently working towards malaria elimination [1], many of which are on track to eliminate by 2020 [2]. Reductions in the global malaria burden have been supported by substantial increases in funding between 2000 and 2011 by domestic governments and bi- and multi-lateral donors, but most notably from the Global Fund to Fight AIDS, Tuberculosis and Malaria (Global Fund). Disbursements to malaria-endemic countries from bi- and multi-lateral donors increased during this time period from US$100 million in 2000 to US$1.84 billion in 2011 [3]. Despite this increase, global aid investments have leveled off since 2011 and countries are working to maintain their progress with fewer external resources [4].

In comparison to countries with higher burdens of malaria, global financing for malaria-eliminating countries is much lower and potentially more tenuous, particularly under the Global Fund's New Funding Model (NFM). Between 2006 and 2010, the proportion of disbursements from domestic and international donor funding received by malaria-eliminating countries accounted for just 17% of the global funding for malaria [5]. Most investments in malaria elimination have typically come from domestic governments, limited bilateral donors such as Australia, and most significantly, from donors channeled through the Global Fund [5]. Although 41% of all persons currently at risk (PAR) for malaria live in the 34 malaria-eliminating countries [3], only 7% of total malaria Global Fund grants go to eliminating countries. For about half of these countries, Global Fund funding accounts for between 35% and 88% of total malaria expenditures [3]. The majority of international funding for malaria has typically been allotted to higher malaria burden countries where control is crucial but elimination may not yet be possible [5].

With the Global Fund's NFM—aimed at increasing “value for money” by focusing on high burden, low income countries—there is a major structural shift that may disproportionately affect malaria-eliminating countries, which are low burden and often middle-income. Under the NFM, fewer total resources coupled with a prescriptive allocation method may leave eliminating countries (among those still eligible) without the funds necessary to continue their elimination efforts. This may penalize eliminating countries for past successes. For countries that have already committed to malaria elimination, disruption of malaria programs risks malaria resurgence [6] that would erase the benefits of past malaria investments.

To guide resource mobilization efforts for filling future funding gaps for the 34 malaria-eliminating countries, this study aims to: 1) assess past trends in donor malaria funding, particularly from the Global Fund, and 2) estimate the future funding needs to achieve malaria elimination by each country's stated target date and prevent reintroduction through 2030. Implications for meeting projected funding needs in anticipation of the Global Fund's NFM are highlighted.

## Data and Methods

This analysis focused on the 34 malaria-eliminating countries listed in Table 1, most recently classified in 2013 [2]. Table 1 also lists each country's stated or assumed target malaria elimination date (as of August 2013). Stated national malaria elimination goals were used in all possible cases where countries have made such a declaration. For countries that do not have a stated national target, we made several assumptions. Argentina and Paraguay do not have national goals, but because only 18 and 10 cases per year were reported in 2011, respectively [3], we assumed that they will reach zero transmission by 2015. Belize, Costa Rica, Mexico, Nicaragua, and Panama do not have stated national elimination goals, but have announced a collective regional elimination goal of 2020 in their recent Global Fund grant for the Elimination of Malaria in Mesoamerica and Hispaniola (EMMIE) (one of two new regional initiatives funded through the Global Fund from 2014 to 2016, which awards US$10 million to ten countries in Latin America through a cash-on-delivery model. The other initiative is the Regional Artemisinin Resistance Initiative (RAI) in the Mekong Region anticipated to receive US$100 from the Global Fund to battle artemisinin resistance in Cambodia, Laos, Myanmar, Thailand, and Vietnam). [7] Based on progress and current epidemiology of the remaining countries that either do not have a stated elimination goal (Iran, the Democratic People's Republic of Korea (DPRK), and Thailand) or countries with only provincial or subnational goals (the Philippines, Solomon Islands, and Vanuatu), the authors anticipate elimination in most by 2025, and all by 2030. Thus, for this analysis, the conservative assumption of a 2030 elimination target will be used for these six countries.

**Table 1:** 34 malaria-eliminating countries, sorted by identified national target malaria elimination goals.

Country	WB Country Code	National elimination goal
Azerbaijan^1^	AZE	2014
Solomon Islands*	SLB	2014
Sri Lanka	LKA	2014
Vanuatu*	VUT	2014
Algeria	DZA	2015
Botswana	BWA	2015
Cape Verde	CPV	2015
Kyrgyzstan	KGZ	2015
Republic of Korea	KOR	2015
Sao Tome and Principe	STP	2015
Saudi Arabia	SAU	2015
Swaziland	SWZ	2015
Tajikistan	TJK	2015
Turkey	TUR	2015
Uzbekistan	UZB	2015
Bhutan	BTN	2016
South Africa	ZAF	2018
Belize^2^	BLZ	2020
China	CHN	2020
Costa Rica^2^	CRI	2020
Dominican Republic^2^	DOM	2020
El Salvador^2^	SLV	2020
Malaysia	MYS	2020
Mexico^2^	MEX	2020
Namibia	NAM	2020
Nicaragua^2^	NIC	2020
Panama^2^	PAN	2020
Philippines*	PHL	2020
Vietnam	VEN	2030
Argentina	ARG	NNG
Democratic People's Republic of Korea	PRK	NNG
Iran (Islamic Rep.)	IRN	NNG
Paraguay	PRY	NNG
Thailand	THA	NNG

*Provincial goals, therefore 2030 is assumed for national goal.

NNG: No National Goal. If no national elimination goal is identified, 2030 is assumed unless otherwise noted in methodology.

1 Azerbaijan's national goal for elimination was by 2013; however authors have assumed 2014 since no declaration of elimination has been reported.

2 Elimination goal of 2020 declared under the EMMIE regional initiative.

### Data sources

Country-level data for Gross National Income (GNI) per capita (Atlas Method) was sourced from the World Bank (http://data.worldbank.org/) for the years 2000 to 2010. Disbursement amounts by donor source for international and domestic financing for malaria data and annual parasite incidence (API), where API  =  confirmed cases per year/population at risk x 1000 [8], extracted and collated for the years 2005 through 2010 where available from the World Health Organization (WHO) *World Malaria Report 2012* and WHO Statistical Information System (http://www.who.int/whosis/en/) respectively. Population data came from the United Nations Population Division (https://data.undp.org/). Malaria Atlas Project (MAP) (http://www.map.ox.ac.uk/) estimates of PAR [9] were used to calculate quantities of interventions that would apply to the population at risk for malaria, such as diagnostics, monitoring and evaluation, surveillance, detection and outbreak response. As the current international standard, PAR served as a denominator for surveillance in order to express risk over the entire population to make sure no cases arise. Because PAR estimates were used to calculate interventions that are employed during elimination and through prevention of introduction, PAR was assumed to remain constant, only adjusted for population growth. WHO malaria incidence rates were used to calculate intervention coverage needs, such as for treatment, and are assumed to decline over time as malaria is progressively eliminated (described below). Drug prices for Coartem were taken from the manufacturer's publicly stated retail/wholesale price [10], and prices for chloroquine and primaquine were sourced from a joint WHO and UNICEF sourcing and price report [11]. Prices for microscopy were also taken from a WHO report on determining cost effectiveness of malaria diagnostics (2006) and assumed to have a 10% quality assurance rate [12]. G6PD test (via Trinity FST) costs were collected from PATH [13]. Median Long-lasting insecticidal net (LLIN) costs were taken from a UNICEF LLIN price report [14].

### Data analysis

Two types of analyses were conducted. First, we describe the general association between financing and health progress from 2005 to 2010 by assessing time trends in malaria burden vis a vis API and the amount of financing for malaria received internationally, and specifically from the Global Fund. Under the NFM, GNI per capita is the income level indicator used to determine the allocation portfolio for each country. As an indication of an individual countrie's capacity for increased domestic financing for future malaria activities, GNI per capita average annual growth rate between 2000 and 2011 was plotted against the 2011 GNI per capita income level.

Second, we estimated the costs needed to eliminate malaria and prevent reintroduction through 2030. The methodology for cost projections was adapted from models for malaria control in Kizewski et al. [15]. The interventions and underlying assumptions in the deterministic model were adjusted to reflect interventions and program measures required for malaria elimination and prevention of reintroduction. A subset of interventions, such as bed nets and community health workers, are carried out until country reported target year of elimination, where zero locally transmitted cases occur, while surveillance and response activities, and diagnostics and treatment are continued after elimination has been reached in order to detect and treat any outbreaks. While costs to maintain prevention of reintroduction may continue long after elimination, we end our cost projections in 2030, the year in which all 34 malaria-eliminating countries are assumed to eliminate in this study. Table 2 summarizes the interventions included in the model and the duration of the interventions assumed. The basis for estimating the coverage for intervention accords to one of two factors:

**Table 2:** Interventions included in the cost projections.

Type of Intervention	Duration	Calculated based on PAR or Incidence?
*Plasmodium falciparum* Treatment	Through 2030	Incidence
*Plasmodium vivax* Treatment	Through 2030	Incidence
Severe and Complicated Malaria Treatment	Through 2030	Incidence
IPT	Through 2030	Adjusted PAR* % Women * fertility rates
Diagnostics	Through 2030	PAR
LLINs at 30% coverage	Through elimination year	PAR
CHWs	Through elimination year	PAR
Monitoring and Evaluation	Through 2030	PAR
Detection/Interdiction (including indoor residual spraying)	Through 2030	PAR

- 1) coverage for the estimated population at risk, which is assumed to be constant, adjusted with average population growth rates; or
- 2) coverage for malaria incidence, which is assumed to decline over time exponentially by I(t)  = I*e^∧^-λt (where *I* =  incidence at time *t* with λ = 0.2), asymptomatically approaching zero between 2014 and 2030 such that incidence never reaches zero.

Maintaining minimal incidence levels past the elimination year was intentionally done to mimic low levels of imported cases, affecting outbreak response measures such as maintaining drug stockpiles. Because malaria incidence varied widely across eliminating countries and from year-to-year, we sought to reduce this variability and limit the extent of mis-measurement by grouping incidence rates according to the 2011 median annual cases per 1,000 PAR, which clustered around three central tendencies. The majority of the countries (n = 28) were included in cluster 1 with a median of 0.63 API. A median of 14.29 API was used for Belize, DPRK, and Republic of Korea. A median of 111.79 API was used for Sao Tome and Principe, the Solomon Islands, and Vanuatu. Incidence was then assumed to decline exponentially from each clusters' starting median cases per 1,000 PAR. Additional information can be found in Figure S1 of S1 File.

Other model assumptions are as follows:

- Commodity costs are constant 2013 prices in US dollars.
- Existing country capacity, tools, interventions and technologies are assumed for delivering future interventions, but allowances for training on emerging new tools or strategies (e.g. point-of-care diagnostics or more efficient sampling techniques) introduced during the shift from elimination to prevention of reintroduction is included under surveillance.
- Treatment for complicated *Plasmodium falciparum* is assumed for 3% of annual incidence for children and 1% of annual incidence for adults; treatment for complicated *Plasmodium vivax* are not included as such cases are rare [16]. “Severe” or “complicated” malaria includes severe anemia, cerebral manifestations and/or respiratory distress syndromes.
- Intermittent Preventive Treatment (IPT) is restricted to the proportion of women aged 15–49 (according to fertility rates [17]) and for countries which reportedly use IPT as an intervention (i.e. Cape Verde, Namibia, and São Tomé and Príncipe).
- As treatment costs are calculated based on exponentially declining incidence, treatment costs will decrease, but never reach zero even in the absence of cases to account for drug stockpiles needed for rapid outbreak response.
- 30% of PAR [18] is assumed to receive LLINs until the elimination target year is reached and discontinued thereafter; delivery and replacement costs are included. LLIN usage is typically low in low-transmission settings and targeted around hotspots, which is thought to lead to malaria elimination more efficiently than blanket coverage. The theory of over-dispersion states that a small proportion (20%) of the population is responsible for the majority (80%) of transmission [17]. Thus, a 30% LLIN coverage was conservatively estimated for all 34 countries.
- Community health workers (CHWs) were included in lower income countries at a rate of 0.5 per 1,000 PAR, with a set stipend amount at 1/6^th^ of the country's minimum wage for salary through to elimination year. [15]
- Diagnostics, monitoring and evaluation, and detection and interdiction (a package of interventions for strategic disruption of transmission including hotspot detection and outbreak response, indoor residual spraying, equipment, and staff training) are calculated based on constant PAR through 2030 to maintain surveillance and response activities after elimination is achieved. Microscopy is assumed to be the main diagnostic method in low burden areas and includes 10% quality assurance for monitoring and evaluation. Hotspot detection, outbreak response and IRS costs (e.g. equipment and training) are included in detection and interdiction.

Additional details on methodology and cost model assumptions can be found in S1 File.

### Cost projections sensitivity analysis

Because the results of the costing model may be sensitive to key assumptions, sensitivity analysis was performed on two main parameters. First, we applied two stepwise declining coverage rates (estimating low coverage and estimating high coverage) to the main cost driver, LLINs, and compared results against the main model. Percent coverage rates for the “low coverage scenario” are difficult to estimate given the lack of evidence in low transmission settings and the sensitivity of coverage due to changes in funding allocations and potential net disbursement fluctuations. Empirical evidence from Sri Lanka suggests the coverage ranges from 5% to 35% [19]. Therefore the conservative range of 15% to 50% was used for the “low coverage scenario”: 50% coverage of PAR was assumed when countries were more than 10 years away from reaching elimination, 30% when countries were five to nine years away from reaching elimination, and 15% coverage when countries were within five years away from elimination.

The “high coverage scenario” assumed 80% PAR coverage when countries were more than 10 years from elimination, 50% for when countries were five to nine years from elimination, and 30% coverage when countries were within five years from elimination. “High coverage scenario” coverage rates are based on averaged WMR data for all malaria endemic countries [3].

Second, main model cost estimates were compared to a version adjusted by country-specific remoteness and incapacity indices (RII), such that RII  =  *a^1/2^p^−3/2^*, where *a* represents the area size of a country and *p* is the population [20]. The RII adjusts costs upward in places that may require more expensive delivery mechanisms to reach relatively more dispersed populations at risk given infrastructure and transportation challenges. Conversely, RII adjusts costs downward in places where service delivery may be more efficient due to increased population density or accessibility.

## Results

Between 2005 and 2010, the majority of malaria-eliminating countries experienced both an increase in total malaria funding, which includes national and external funding, and a decrease in API (see Table 3). Over 60% (n = 21) of countries have reduced API that concurrently occurred with increases in total malaria funding. As the largest financer for malaria, the Global Fund has provided substantial funding to 14 of the malaria-eliminating countries in this quadrant. About 20% (n = 7) have experienced greater than a 60% reduction in API despite a decrease in funding. Finally, 18% (n = 6) of malaria-eliminating countries have steady or increased API despite increased funding from all sources over this time period. There have not been any countries where API has increased while funding has declined. Belize and South Africa were both outliers in which total malaria financing from both domestic and external sources decreased by 302% and 524%, respectively, with reductions in API of 85% and 2%; neither country has received direct support from the Global Fund.

**Table 3:** Change in total malaria funding, percentage of Global Fund funding, and API between 2005 and 2010.

		Total malaria financing (2005–2010)			
		Decrease		Increase	
API	Decrease	Country	% from GF	Country	% from GF
**(2005–2010)**		Uzbekistan	80%	China	100%
		Nicaragua	65%	Tajikistan	88%
		Argentina	0%	Philippines	84%
		Belize	0%	Kyrgyzstan	76%
		Cape Verde	0%	Bhutan	68%
		Panama	0%	Vietnam	56%
		South Africa	0%	Namibia	55%
				Sao Tome and Principe	46%
				Sri Lanka	42%
				Solomon Islands	35%
				Vanuatu	25%
				Swaziland	22%
				Azerbaijan	21%
				Iran	12%
				Algeria	0%
				Costa Rica	0%
				El Salvador	0%
				Mexico	0%
				Paraguay	0%
				Saudi Arabia	0%
				Turkey	0%
	**Increase**			Thailand	66%
				Democratic People's Republic of Korea	41%
				Dominican Republic	16%
				Botswana	0%
				Malaysia	0%
				Republic of Korea	0%

Notes: Countries are clustered in the top right quadrant indicating higher reductions in API and an increase in total funding for malaria. Countries in the lower right quadrant have either stagnating or increasing APIs despite increases in funding, likely due to importation issues. In the upper left quadrant, countries have reduced APIs, with a commensurate decrease in funding.

Source: Data taken from the World Health Organization's 2011 World Malaria Report Annex 2 for total funding for malaria includes country reported government and external funding for the period of 2005–2010, and does not include “Contributions reported by donors”. Additionally, due to sparse data, the percent increase in total funding for malaria was calculated for the following countries during the respective time period: Algeria (2008–2010); Belize (2005–2009); Dominican Republic (2007–2010); El Salvador (2005–2009); Malaysia (2007–2010); Nicaragua (2006–2010); South Africa 2007–2010; Swaziland (2007–2010); Uzbekistan (2005–2009).

**Figure 1:**
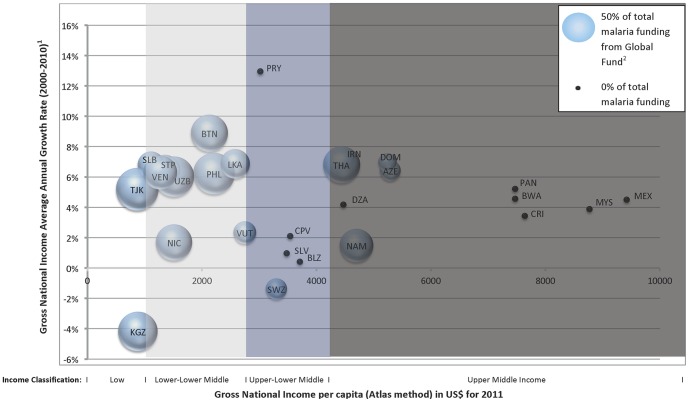
Average annual growth rate in Gross National Income per capita between 2000 and 2010 by Gross National Income per capita for 2011. Notes: The Global Fund income categories are based on the World Bank (Atlas Method) Income Classifications. Lower middle income countries are further divided into two groups: lower-lower middle income countries and upper-lower middle income countries based on the midpoint of the GNI per capita range of the lower middle income category. Classifications are as follows: low income, $1,025 or less; lower-lower middle income, $1,026–$2,530; upper-lower middle income $2,531–$4,035; upper middle income, $4,036–$12,475. GNI per capita average annual growth data for the Democratic People's Republic of Korea was unavailable. Data for China is unreliable, reporting 100% of malaria funding from the Global Fund, and therefore removed. High income countries—Korea, Saudi Arabia, and Turkey—are not shown. ^1^Data obtained from the World Bank. If information was not available for 2010, data from the most recent year available was used. ^2^Data taken from the World Health Organization's 2011 World Malaria Report Annex 2 for the period of 2005-2010, not including contributions reported by donors. Bubble legend to scale.

Three malaria-eliminating countries are classified as high income countries, 14 as upper middle income countries, 14 as lower middle income countries (eight are lower-lower middle income (LLMI), six are upper-lower middle income (ULMI)), and three are lower income countries (DPRK, Kyrgyzstan, and Tajikistan). Figure 1 displays the average annual growth in GNI per capita by each country's 2011 GNI per capita with the bubble size representing to scale the percent of total malaria funding coming from the Global Fund. Kyrgyzstan and Tajikistan, with GNI per capita annual growth at −4% and 5% respectively, have been dependent on Global Fund financing, which has comprised 76% and 88% of total expenditures. As Global Fund funding shifts to higher burden/lower income countries, low burden Global Fund-dependent countries such as Kyrgyzstan and Tajikistan may be in danger of significantly reduced future malaria funding. While DPRK has also relied on the Global Fund for 41% of malaria financing, GNI per capita annual growth rate data was unavailable and could not be plotted. Just over half of the malaria-eliminating countries that are Global Fund-supported are categorized as either LI or LLMI. LLMI countries have experienced between 1% and 9% annual GNI per capita annual growth, and all have received between 35% and 84% of their malaria funding from the Global Fund (2005–2010). UMLI countries have experienced less economic growth, ranging from −1% to 2%, with the exception of Paraguay, where per capita GNI growth was 13% and Global Fund support has been low. Three of the lower middle income countries (Nicaragua, Swaziland and Vanuatu) have lower GNI per capita annual growth (ranging from −1% to about 2%), which suggests it may be more difficult for their governments to fill potential funding gaps created by the NFM. Other Global Fund dependent countries that have seen higher GNI per capita annual growth rates may have an easier time shifting away from Global Fund funding and increasing domestic financing for malaria. Of the ULMI countries, only Vanuatu and Swaziland have received grants from the Global Fund, accounting for about a quarter of their total malaria expenditures each. The upper-middle income countries are to the far right on the graph (i.e. Panama, Belize, Costa Rica, Malaysia, and Mexico) and have not received or have not been eligible for any funding from the Global Fund.

**Figure 2:**
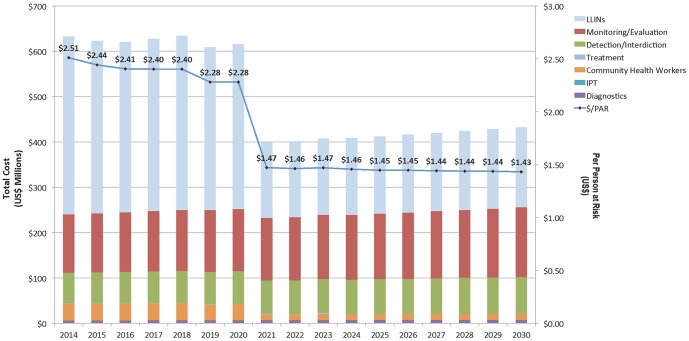
Estimated costs for malaria elimination and prevention of reintroduction in the 34 malaria-eliminating countries, 2014–2030. Notes: The decrease in 2020 is due to a number of countries reaching their national target elimination years, at which time, based on our model assumptions, certain interventions cease (CHWs, LLINs). Other interventions, such as treatment continue, decline by reducing coverage levels per declining incidence. Estimated costs for 2021 to 2030 include all projected expenditures for maintaining elimination interventions thorough 2030 for DPRK, Iran, the Philippines, Thailand, Solomon Islands, Vanuatu, and Vietnam. This period also includes expenditures for prevention of reintroduction interventions among countries that have eliminated prior to 2021. Prices are in 2013 USD$.

Results of program cost projections from 2014 through 2030 are shown in Figure 2. Based on the model calculations, approximately US$8.5B will be needed over the entire period to sustain program activities for elimination and prevention of introduction in the 34 malaria-eliminating countries. Standardized by per person at risk per year, this translates to $2.51 in 2014, declining to $2.28 in 2020, dropping to $1.47 in 2021 (when 22 of the 34 countries reach their elimination target year), and reducing further to $1.43 by 2030.

At 30% blanket coverage, LLINs are the main cost driver, representing 51% of overall costs between 2014 and 2030. Over time, however, LLIN costs decline as countries reach their elimination targets. The model's second and third major cost drivers, monitoring/evaluation and detection/interdiction respectively, each increase by approximately 20% over time in order to maintain elimination and prevent reintroduction of malaria.

**Figure 3:**
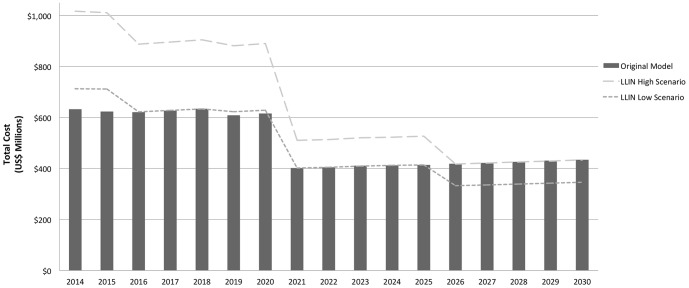
LLIN adjusted costs to eliminate malaria in 34 countries, 2014–2030. Notes: Higher LLIN estimates occur from 2014 to 2020 are associated with higher coverage rates for countries further from elimination years in both scenarios (80% for “high coverage scenario” and 50% for “low coverage scenario”). As elimination year nears, countries move into the next coverage tier of both scenarios (50% for “high coverage scenario” and 30% for “low coverage scenario”). For all endemic countries between the years of 2026 and 2030, coverage rates are at their lowest (30% for high coverage scenario and 15% for low coverage scenario).

The sensitivity analysis around LLIN ranges can be found in Figure 3. In both tiered scenarios, LLIN costs are above those of the main model for the first six years (2014–2020), then decrease between 2020 and 2021, and once more decline between 2025 and 2026 before leveling off until 2030. This pattern reflects each country's declining LLIN coverage rate as they move toward elimination. The overall cumulative costs for both scenarios range from approximately US$8.3B in the “low coverage scenario” to US$11.2B in the “high coverage scenario”.

**Figure 4:**
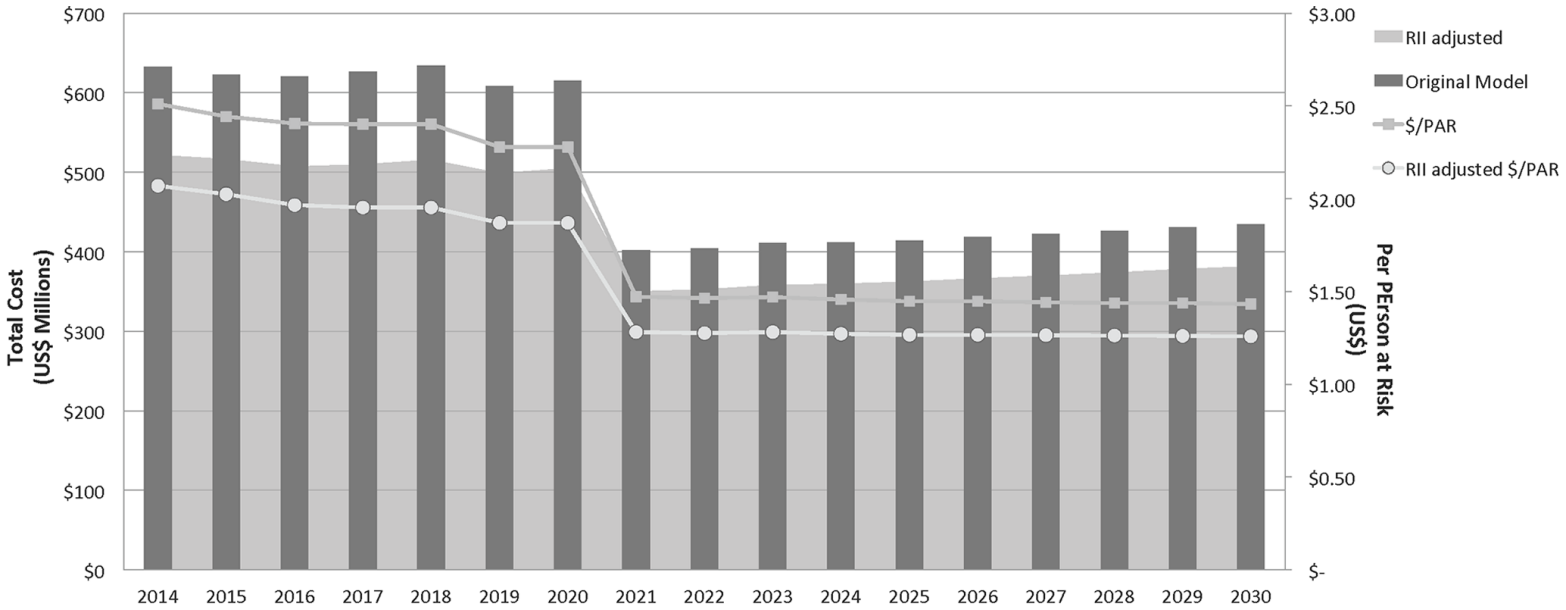
Remoteness and Incapacity Indices (RII) adjusted costs to eliminate malaria in 34 countries, 2014–2030. Estimated overall and per PAR costs from the original model are compared over time to RII adjusted costs. RII adjusted rates are about 15% lower than the original model.

Figure 4 contrasts the main cost model with the RII-adjusted model. The application of the RII decreased overall costs by 15% to US$7.2B, with corresponding lower $/PARs ranging from $2.07 in 2014 to $1.26 in 2030.

## Discussion

Malaria-eliminating countries have reduced their overall malaria burden since 2005, while simultaneously benefiting from increases in global financing for malaria. Even though the Global Fund is the largest direct financial contributor to malaria-eliminating country programs, disbursed amounts for malaria-eliminating countries have only accounted for a fraction of all monies going toward malaria. While not sufficient, this amount has been necessary for financing programs that have scaled up interventions and ultimately reduced cases and deaths. Under the NFM, this financial support is anticipated to decrease when the main funding focus shifts towards higher burden countries [21].

As we have estimated, maintaining basic interventions to reach elimination and prevent reintroduction in the 34 malaria-eliminating countries will require an average of $1.84 per at-risk person per year between 2014 and 2030, about US$8.5B overall. This estimated amount falls within the range of estimates from both sensitivity analyses with an upper limit of US$11.2B from the “high coverage scenario” of the LLIN sensitivity analysis to the lower limit of US$7.2B for the RII adjusted scenario. Trends in drug and insecticide resistance or changes in vector feeding habits may necessitate additional interventions and increased coverage rates beyond what has been included in this model in order to achieve elimination. However, as the costs of preventing reintroduction are overall estimated to be lower than the elimination phase, investing money up front to countries that can eliminate malaria will free up resources for other endemic countries.

Some higher income countries have either never or minimally relied on Global Fund to finance malaria activities (e.g. Algeria, Argentina, Belize, Costa Rica, the Republic of Korea, Malaysia, Saudi Arabia, South Africa, and Turkey) while others have “graduated” and are no longer eligible (e.g. Azerbaijan, China, and Mexico). In conjunction with increasing/positive annual growth rates, these countries may be relatively better-off and be able to continue program activities based on domestic financing alone. For many LLMI countries, higher GNI per capita average annual growth rates signal greater potential for boosting government spending on malaria, but any increases are unlikely to fulfill the gap needed to successfully continue elimination efforts. In particular, over a third of the malaria-eliminating countries are categorized as LI, LLMI or ULMI, and rely on at least 20% of Global Fund assistance, however, are at risk of decreased support from the Global Fund due to their lower burden status. Of these lower income countries, those with lower GNI per capita average annual growth rates and a larger dependence on Global Fund funding may be less likely to compensate for decreased international funding with domestic resources. Cost-sharing requirements per the NFM may help to facilitate and encourage the shift toward greater domestic financing responsibility; however, radical substitution of funding sources is unlikely to occur quickly. Furthermore, using GNI per capita as the parameter by which the Global Fund assesses a country for allocations overlooks actual domestic usage of funds and warrants a more equitable assessment government health spending for their allocation methodology.

There is also the threat of governments shifting attention as countries move closer to malaria elimination. As malaria is no longer seen as a major threat, resources may be diverted to more pressing budget items and competing priorities. Such was the case with Sri Lanka, which in 1963 almost achieved elimination and then discontinued their malaria program. Shortly thereafter, a devastating resurgence occurred, with more than 400,000 cases reported [22]. Since the resurgence episode, Sri Lanka has once again been able to interrupt local transmission and is actively preventing reintroduction, but it has taken them decades to do this [23]. As experienced in Sri Lanka and numerous other countries, redirecting funds away from malaria programs may lead to costly resurgence events [6].

While a high proportion of the Global Fund malaria portfolio will be allocated to the higher burden, lower income countries, investments should place more weight on the malaria resurgence potential and success should not be penalized. This is particularly important for countries that are moving up income levels and therefore may be entitled to less funding under the predefined income level and disease burden-based country bands of the NFM.

New, more efficient strategies and creative leveraging of resources will be needed to further close potential funding gaps, including increasing financial commitments from private sector donors, domestic governments, innovative financing mechanisms, or collaborative regional financing mechanisms [5]. Efforts to document alternative financing models have been undertaken by Kumar et al. in the Financing for Malaria Elimination report. In particular, regional initiatives may help to hold countries accountable for progress in addition to addressing cross-border transmission and importation risks. With the implementation of the NFM, the Global Fund has taken the encouraging first steps to leverage resources by imposing requirements for increased domestic contributions and catalyzed two new regional initiatives for malaria elimination.

### Limitations

The results of this study should be interpreted in light of several caveats. GNI per capita, the indicator used to analyze economic status by the Global Fund and consequently used in this study, may be too simplistic a measure which does not truly assess capacity for domestic funding. Even if a country has a high GNI per capita, priorities may allocate funding to areas that may or may not be health related. More work is needed for new ways to evaluate economic status such that equity is preserved and available domestic financing resources to achieve elimination are better assessed.

The rate of incidence case decline was assumed to be exponential and unrelated to the level or intensity of intervention. Current malaria model simulations are not yet calibrated for low transmission settings although efforts to do so are underway. Intervention costs are estimated based on individual inputs and potential efficiencies across interventions were not accounted for. Estimates projected for island nations, such as the Solomon Islands and Vanuatu, are likely to be lower than what would actually be needed for two main reasons. First, both islands have only declared sub-national goals, thus additional scale up, which has not been incorporated into this model, is likely needed. Second, delivery of interventions and commodities for island nations tend to be more costly due to accessibility and geography, which were not accounted for in the main model's prices. While the RII was applied in sensitivity analysis to adjust for these factors, this measure may also underestimate delivery costs in other countries (e.g. China, Mexico, South Africa).

Where possible, country-specific data were used, such as for population, PAR, population growth rates, fertility rates, CHW salaries, incidences of *Plasmodium falciparum* and *Plasmodium vivax*. However, in many cases, reliable data was not available. Blanket estimates were used for other parameters, such as coverage rates, commodities, warehousing and distribution costs, and trainings. Severe and complicated infections, assumed at a blanket coverage of 3% for children and 1% for adults is also likely overestimated as percentages were taken from endemic country literature. However, as treatment costs only account for about 1% of total estimated costs, any overestimation would not be significant. Cost projections, therefore, should more appropriately be interpreted as an overall estimate for all 34 malaria-eliminating countries and used as a guide for countries to inform individual assessments.

Inherently, long-term estimates contain many uncertainties. Unforeseen circumstances, changes in political priorities, or a decrease in financing may prohibit a country from achieving elimination by its target year and leave the country at risk for a costly resurgence. Because of the indeterminate nature of these events, only known goals and interventions were considered for this analysis.

### Conclusion

External funding, particularly from the Global Fund, has been a sizable portion of funding for some countries striving to achieve elimination and it is important to maintain these funding resources or increase financing from domestic or alternative sources. Increased advocacy efforts are needed to sustain resources from the Global Fund, maintain political will, and increase domestic and other international funding sources. Additional research should be conducted to improve Global Fund allocation methods by assessing domestic resources earmarked for malaria elimination and ensuring equity is preserved.

Malaria elimination and prevention of reintroduction activities should be perceived and adopted as a necessary recurring investment, such as with vaccinations—a routine program that prevents disease or death even if none exists at that time. Given the expected gap between country needs and 2014 Global Fund allocations, many countries may have to take steps to increase their domestic financing for malaria elimination programs. This may include providing better data to governments to strengthen evidence, furthering research on costing and financing gaps, increasing advocacy activities, or creating efficiencies with other disease responses.

## Supporting Information

S1 File **Malaria Elimination Costing Model: Assumptions.** Figure S1, Clustered incidence declines, based on 2011 incidence per PAR. Table S1, Median annual cases per 1,000 PAR. Table S2, Coartem Price List, 2009. Table S3, Chloroquine Price List. Table S4, Primaquine price list.
(DOCX)
