# Supplementary material for: Costs of Eliminating Malaria and the Impact of the Global Fund in 34 Countries
Source: PLoS One. 2014 Dec 31;9(12):e115714. doi: 10.1371/journal.pone.0115714 (PMC4281070; doi:10.1371/journal.pone.0115714)
Supplement: S1 File — Malaria Elimination Costing Model: Assumptions. Figure S1, Clustered incidence declines, based on 2011 incidence per PAR. Table S1, Median annual cases per 1,000 PAR. Table S2, Coartem Price List, 2009. Table S3, Chloroquine Price List. Table S4, Primaquine price list. (DOCX) [file pone.0115714.s001.docx]

**File S1: Malaria Elimination Costing Model: Assumptions**

Cost estimates of interventions through elimination and prevention of reintroduction for 34 malaria-eliminating countries until 2030 are calculated based one of two factors:

1. Coverage for the estimated population at risk, or
2. Coverage for malaria incidence.

The Malaria Atlas Project (MAP) 2010 estimates of population at risk are kept constant through 2030, adjusted only for average population growth rates. Specific interventions calculated based on PAR are assumed to be discontinued when elimination is reached (i.e. LLINs, and the use of community health workers (CHWs)). Diagnostics, monitoring and evaluation, and detection and interdiction are assumed to continue at a constant rate based on PAR through elimination to 2030 as prevention of reintroduction interventions.

WHO estimates of incidence are assumed to decline exponentially. A simple exponential decline was used: I(t)=I*e^-λt (where *I*=incidence at time *t* with λ=0.2). Maintaining minimal incidence levels post elimination date was intentionally done to mimic low levels of imported cases post elimination date and to maintain drug stockpiles for rapid outbreak response. Thus, treatment for both uncomplicated and severe cases of *Plasmodium falciparum* and *Plasmodium vivax*, as well as intermittent preventive treatment (IPT) are based on declining incidence rates and will continue through 2030.

Countries’ 2011 median incidence rates were found to cluster around three central tendencies and countries were grouped accordingly to account for varying endemicities. Twenty-eight countries were included in cluster 1 with a median of 0.63 annual cases per 1,000 PAR, cluster 2 had a median of 14.29 annual cases per 1,000 PAR (Belize, the Democratic People’s Republic of Korea (DPRK), and Republic of Korea), and a median of 111.79 annual cases per 1,000 PAR was used for cluster 3 (Sao Tome and Principe, the Solomon Islands, Vanuatu). Incidence was assumed to decline exponentially toward 2030 from each clusters’ starting median incidence. For countries in cluster 1, incidence is close to zero, and thus reductions in incidence will be minimal between 2014 and 2030. In contrast, cluster 3 countries have a higher starting incidence and therefore will have a steeper decline in cases over time. Median estimated annual cases per 1,000 PAR are shown in Table S1, and decline rates for each cluster are shown in Figure S1.

**Interventions**:

**1. Basic treatment for uncomplicated cases**

Treatment costs are determined by the 2011 incidence of *Plasmodium falciparum* and *Plasmodium vivax* from 2012 World Malaria Report Country Profiles [1]. The only exception was Paraguay, where the World Malaria Report listed 100% *Plasmodium falciparum* and no *Plasmodium vivax.* Since *Plasmodium vivax* is known to occur, information from the Centers for Disease Control and Prevention was used instead [2].

Distribution and warehousing: An additional ten percent of total commodity costs per year were assumed for distribution costs. For warehousing, an additional $3,000 per 100,000 courses of treatment per year were assumed [3].

a. *Plasmodium falciparum* cases:

All cases are assumed to be treated with either artemisinin-based combination therapies (ACTs) for *Plasmodium falciparum* or chloroquine and primaquine for *Plasmodium vivax,* with dosing dependent on age. ACT costs do not assume a subsidy. To simplify the analysis, Coartem (Novartis) was assumed to be the front-line drug of choice for all countries (see Table S2). Prices have declined over the years under a tiered pricing scheme differentiating commercial from public buyers. The most recent price decrease occurred in 2009 [4].

b. *Plasmodium vivax* cases

Chloroquine (100mg base at $0.005 per tablet, reported by a joint WHO and UNICEF sourcing and price report) is the assumed treatment for *Plasmodium vivax* [5]. For adults, a loading dose of 600mg, followed by 300mg at 6, 24 and 48 hours (1,500 mg total) is assumed [6]. For pediatric doses, a loading dose of 10mg/kg, followed by 5mg/kg at 6, 24 and 48 hours (25mg/kg total) is assumed (see Table S3). For treatment with Primaquine, a 7.5mg base at $0.005 price per tablet was assumed as per WHO [5]. The adult dose is 30mg base per day over 14 days; the pediatric Dose is 0.5mg base/kg per day over 14 days (not recommended in <1 year olds) (see Table S4) [6]. For G6PD testing, the use of the most commonly used, mid-priced, commercially available NADP-based qualitative test, Fluorescent Spot Test by Trinity, was assumed at $4.35 per test [7].

**2. Intermittent Preventive Treatment (IPT) for Pregnancy**

We assume IPT only for selected countries that have indicated to WHO that it is part of their national malaria control strategy: Cape Verde, Namibia, and São Tomé and Príncipe. Sulfadoxine-Pyrimethamine (SP) is the drug of choice: three tablets four times during pregnancy (1,500 sulfadoxine and 75 pyrimethamine per dose). Each tablet is assumed to cost $0.02. Treatment guidelines for IPT derive WHO Global Malaria Program [8] and the median price derives from the WHO [5].

**3. Diagnostic Testing (during passive and active case detection)**

WHO estimates malaria microscopy from a report on determining cost effectiveness of malaria diagnostics to range between $0.32 and $1.27 with a best estimate of $0.53 per blood smear [9]. The number of diagnostic tests utilized per year is assumed to be double the rate of actual malaria incidence among age groups.

**4. Long-lasting Insecticidal Nets (LLINs)**

We assumed that 30% of PAR receives LLINs on a regular cycle, with replacement after three years of use. Provision of nets occurs on a staggered cycle, with 40% of nets provided the first year, followed by 30%, 20%, 10% in the following years. This derives from the experience that not all residents are equally accessible. As distribution proceeds into less accessible areas, fewer nets can be distributed per unit of effort. While insecticides may remain active for that long, many nets become comprised with holes before the first year is up.

We used a median price for standard-sized 100 denier or more nets from a UNICEF LLIN price report [10]: 190X180X150cm (LxWxH), deltamethrin or alphacypermethrin at $3.19 each. This price is assumed to be for LLINs delivered to the port of entry. $2.00 per net is assumed for storage and distribution costs as per Kiszewski et al. [3].

**5. Treatment of Severe and Complicated Malaria (*Plasmodium falciparum* only)**

Quinine therapy was assumed to be $29.50 per case [3]. Incidence rate, ranging from 1% of clinical cases in adults to 3% of 1-5 year olds, was assumptions based on a study completed by Oduro et al. [11]. This study found a median rate of 3.4% of severe cases, and a range of 0.4% - 8.3% among children under five. The 0.4% rate was found in children over the age of 4 years while the 8.3% was found in newborns less than twelve months, which supports our assumption of a decline in severity with advancing age. Treatment for complicated *Plasmodium vivax* was not included as such cases are rare.

**6. Community Health Workers**

Community Health Workers (CHWs) are included for all non-high income countries at a rate of 0.5 per 1,000 PAR. CHWs are assumed to be paid a stipend at 1/6^th^ of local annual minimum wage [3].

**7. Monitoring and Evaluation**

Prevalence surveys and similar activities are currently estimated at a flat rate of $5,000 per survey per 10,000 people [3]. Active case detection is also included with microscopy assumed to be the preferred method of diagnosis with a 10% quality assurance. Costs are calculated based on PAR through 2030 for all countries.

**8. Detection and Interdiction with Indoor Residual Spraying**

The pricing assumptions are derived from the epidemic malaria interventions activities priced in Kiszewski et al. (2007) [3]:

***“****The basic package of equipment and supplies was defined on the basis of several successful Global Fund proposals (e.g. Ethiopia, Kenya, Tanzania). The “surveillance package” includes computers and software for health information systems and training in early detection and surveillance methods. This package included one computer (USD 1,500), one printer (USD 300), one voltage stabilizer (USD 300), miscellaneous supplies (USD 200 annually) and computer training (USD 2,000 every 10 years). Computer-related equipment was assumed to have a useful life of five years, and an allowance of 5% of the total cost was added to account for annual maintenance and replacement parts. The total number of units required was assumed to be proportional to the size of the population at risk at the rate of one unit to each 100,000 people living in epidemic-prone areas.*

*The “intervention package” includes sets of equipment, commodities and personnel necessary for conducting IRS. This includes eight 8-liter spray pumps at USD 110 each, plus the cost of international shipment (based on regional average shipment rate mark-ups) and 2% of the equipment cost per year for spare parts. The useful life of a spray pump is assumed to be seven years. One package was assumed to be sufficient to provide services for 100,000 people at risk. Training costs were estimated on the basis of squads of eight spray-men per 100,000 people, with five days of training per squad including per diem and country-specific estimated travel costs for trainees, trainers, and support staff. Materials associated with IRS training cost USD 6.00 per trainee. Training sessions required two trainers and two support staff per squad. After the initial training during the first year, we assumed that a 10% annual attrition rate would require retraining of new spray-men. Ten percent of districts would receive a two-day refresher course every year.*

*The cost of insecticides for conducting IRS was estimated by considering the number of households living at risk of epidemic malaria, assuming an average household size of six people. Pyrethroid insecticides were found to be intermediate in cost between the least expensive (DDT) and the most expensive (carbamates) options among the residual insecticides listed in “Sources and Prices.” Thus, alpha-cypermethrin, a moderately priced pyrethroid, was chosen to represent the typical cost of an insecticide used for IRS in epidemic malaria intervention. An application rate of 50mg per m^2^, and an average 180 m^2^ surface to be sprayed per household was assumed. This led to an estimated cost of insecticide of about USD 9.00 per household.”*

**References**

1. World Health Organization. (2012) World Malaria Report 2012. World Health Organization, Geneva, Swizterland.
2. Tan KR, Steele SF. (2013). Travel Vaccines & Malaria Information, by Country. Available: <http://wwwnc.cdc.gov/travel/yellowbook/2014/chapter-3-infectious-diseases-related-to-travel/travel-vaccines-and-malaria-information-by-country/paraguay#seldyfm533>.
3. Kiszewski A, Johns B, Schapira A, Delacollete C, Crowell T, et al. (2007) Estimated global resources needed to attain international malaria control goals. Bulletin of the World Health Organization 85(8): p623-630.
4. Medicines for Malaria Venture. (2009) Coartem Price List. Available: <http://www.mmv.org/sites/default/files/uploads/docs/news/Coartem_price_list_Aug_2009_0.pdf>. Accessed 15 January 2014.
5. World Health Organization, UNICEF. (2010) Sources and prices of selected medicines for children. Available: <http://www.who.int/medicines/publications/essentialmedicines/Sources_Prices2010.pdf>. Accessed 16 June 2014.
6. Centers for Disease Control and Prevention. (2013). Guidelines for Treatment of Malaria in the United States. Available: http://www.cdc.gov/malaria/resources/pdf/treatmenttable.pdf
7. Centers for Disease Control and Prevention. (2013). Guidelines for Treatment of Malaria in the United States. Available: http://www.cdc.gov/malaria/resources/pdf/treatmenttable.pdf
8. World Health Organization. (2012). Intermittent Preventive Treatment of malaria in pregnancy using Sulfadoxine-Pyrimethamine (IPTp-SP), Updated WHO Policy Recommendation. World Health Organization Global Malaria Program, Geneva, Swizerland.
9. World Health Organization, Western Pacific Region. (2006) Determining Cost Effectiveness of Malaria Rapid Diagnostic Tests in Rural Areas with High Prevalence. Available: <http://www.wpro.who.int/malaria/NR/rdonlyres/0A45DF24-4245-4BF3-96E7-5F1150AF333B/0/DeterminingCostEffectivenessofRDTsinruralareaswithhighprevalence.pdf>
10. UNICEF. (2013) Long Lasting Insecticidal Nets (LLINs) price data. Available: <http://www.unicef.org/supply/files/LLINs_price_transparency_August_2013.pdf>
11. Oduro A, Koram K, Rogers W, Atuguba F, Ansah P, et al. (2007) Severe falciparum malaria in young children of the Kassena-Nankana district of northern Ghana. Malaria Journal 6(1) 96.

**Appendix Figures:**

**Figure S1. Clustered incidence declines, based on 2011 incidence per PAR** [1]


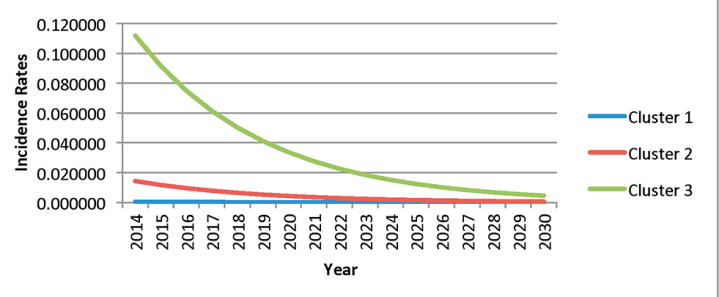


Note: Twenty-eight countries were included in cluster 1 with a median cases per 1,000 PAR of 0.63; a median of 14.29 cases per 1,000 PAR was used for cluster 2: Belize, the Democratic People’s Republic of Korea (DPRK), and Republic of Korea; and a median of 111.79 cases per 1,000 PAR was used for cluster 3: Sao Tome and Principe, the Solomon Islands, Vanuatu. Starting with these initial median APIs, incidence was assumed to decline exponentially toward 2030. For countries in cluster 1, incidence per 1,000 PAR is close to zero, and thus reductions in incidence will be minimal. In contrast, cluster 3 countries have a higher starting incidence per 1,000 PAR and therefore will have a steeper decline in cases over time as they reach elimination.

**Appendix Tables:**

**Table S1: Median annual cases per 1,000 PAR** [1]

| **Country** | **Cases** | **Annual cases per 1,000 PAR** |
| --- | --- | --- |
| **Cluster 1** | | |
| Algeria | 18 | 0 |
| Argentina | 85 | 0.67 |
| Azerbaijan | 59 | 0.13 |
| Bhutan | 757 | 1.71 |
| Botswana | 3913 | 3.73 |
| Cape Verde | 140 | 0.69 |
| China | 12253 | 0.0007 |
| Costa Rica | 132 | 1.36 |
| Dom. Rep. | 4728 | 0.58 |
| El Salvador | 28 | 0.00009 |
| Iran | 2,174 | 1.67 |
| Kyrgyzstan | 4 | 0.02 |
| Malaysia | 16,223 | 0.054 |
| Mexico | 1,443 | 1.16 |
| Namibia | 2,996 | 1.91 |
| Nicaragua | 1,298 | 0.33 |
| Panama | 492 | 1.81 |
| Paraguay | 32 | 1.35 |
| Philippines | 58,976 | 1.1 |
| S. Africa | 17,435 | 1 |
| S. Arabia | 34 | 0.2 |
| Sri Lanka | 2221 | 0.21 |
| Swaziland | 391 | 1.06 |
| Tajikistan | 131 | 0.13 |
| Thailand | 140,844 | 3.41 |
| Turkey | 11 | 0.07 |
| Uzbekistan | 4 | 0.123 |
| Vietnam | 25200 | 0.45 |
| **Median API** |  | **0.63** |
| **Cluster 2** | | |
| Belize | 642 | 26.41 |
| N. Korea | 31,379 | 13.89 |
| S. Korea | 4,346 | 14.29 |
| **Median API** |  | **14.29** |
| **Cluster 3** | | |
| STP | 21,511 | 117.97 |
| Solomon Islands | 65,015 | 111.79 |
| Vanuatu | 25,584 | 96.08 |
| **Median API** |  | **111.79** |

**Table S2: Coartem Price List, 2009**

| **Patient weight (kg)** | **Age in years** | **Price per treatment** |
| --- | --- | --- |
| 5-15 | 0-3 | $0.36 |
| 15-25 | 4-8 | $0.72 |
| 25-35 | 9-13 | $1.08 |
| >35 | 14+ | $1.30 |

Notes: Taken from <http://www.mmv.org/sites/default/files/uploads/docs/news/Coartem_price_list_Aug_2009_0.pdf>.

**Table S3: Chloroquine Price List:**

| **Patient weight (kg)** | **Age in years** | **Max Total Base (mg)** | **Price per Course** |
| --- | --- | --- | --- |
| <10 | <1 | 250 | $0.02 |
| 10-<19 | 1-5 | 475 | $0.02 |
| 19-<24 | 6-7 | 600 | $0.03 |
| 24-<35 | 8-11 | 875 | $0.04 |
| 35-<50 | 12-14 | 1,250 | $0.06 |
| 50+ Adult Dose | 15+ | 1,500 | $0.08 |

Notes: Weight/Age Profile taken from: <http://www.actmalaria.net/files/drugpol_Timor-Leste.ppt> *‎*

**Table S4: Primaquine price list:**

| **Patient weight (kg)** | **Age in years** | **Daily max** | **Total Base (mg)** | **Price per Course** |
| --- | --- | --- | --- | --- |
| <10 | <1 | 0 (not rec) | 0 | $0.00 |
| 10-<19 | 1-5 | 10mg/day | 140 | $0.01 |
| 19-<24 | 6-7 | 12.5mg/day | 175 | $0.12 |
| 24-<35 | 8-11 | 17.5mg/day | 245 | $0.17 |
| 35-<50 | 12-14 | 25mg/day | 350 | $0.24 |
| 50+ Adult Dose | 15+ | 30mg/day | 420 | $0.28 |

Notes: Weight/Age Profile taken from: <http://www.actmalaria.net/files/drugpol_Timor-Leste.ppt> *‎*
